# Oral Delivery of a DNA Vaccine Expressing the *PrM* and *E* Genes: A Promising Vaccine Strategy against Flavivirus in Ducks

**DOI:** 10.1038/s41598-018-30258-3

**Published:** 2018-08-17

**Authors:** Juan Huang, Renyong Jia, Haoyue Shen, Mingshu Wang, Dekang Zhu, Shun Chen, Mafeng Liu, Xinxin Zhao, Ying Wu, Qiao Yang, Zhongqiong Yin, Anchun Cheng

**Affiliations:** 1 Avian Disease Research Centre, Sichuan Agricultural University, Chengdu, 611130 China; 2 Institute of Preventive Veterinary Medicine, Sichuan Agricultural University, Chengdu, 611130 China; 3 Key Laboratory of Animal Disease and Human Health of Sichuan Province, Chengdu, Sichuan 611130 China

## Abstract

A flavivirus, named duck tembusu virus (DTMUV), emerged in China in 2010. This virus has caused great economic losses in the poultry industry in China and may pose a threat to public health. As a safe, efficient and convenient vaccine development strategy, DNA-based vaccines have become a popular approach for both human and veterinary applications. Attenuated bacteria have been widely used as vehicles to deliver heterologous antigens to the immune system. Thus, an efficient and low-cost oral delivery DNA vaccine SL7207 (pVAX1-SME) based on envelope proteins (prM and E) of DTMUV and attenuated *Salmonella typhimurium* aroA^-^ strain SL7207 was developed and evaluated in this study. The prM and E antigen proteins were successfully expressed from the vaccine SL7207 (pVAX1-SME) both *in vitro* and *in vivo*. High titers of the specific antibody against the DTMUV-E protein and the neutralizing antibody against the DTMUV virus were both detected after vaccination with SL7207 (pVAX1-SME). Ducks orally vaccinated with the SL7207 (pVAX-SME) vaccine were efficiently protected from lethal DTMUV infection in this study. Taken together, we demonstrated that prM and E proteins of DTMUV possess strong immunogenicity against the DTMUV infection. Moreover, an oral delivery of the DNA vaccine SL7207 (pVAX1-SME) utilizing *Salmonella* SL7207 was an efficient way to protect the ducks against DTMUV infection and provides an economic and fast vaccine delivery strategy for a large-scale clinical use.

## Introduction

Flavivirus infection is a major public health concern worldwide^1^. Duck tembusu virus (DTMUV) is a member of the genus *Flavivirus* whose incidence exploded in China in 2010^2^. According to recent statistical data, DTMUV can be isolated from a wide range of hosts, including mosquito, goose, chicken, swan, pigeon and sparrow^3,4^. Additionally, the virus spreads rapidly and even transmits in the winter^5^. DTMUV has become a major pathogen of ducks in China^6,7^ and has been reported in almost all the major duck-producing regions of this country^8–10^. The morbidity of ducks infected with DTMUV is high, reaching up to 100%, while the mortality is low, ranging from 5% to 30%^11^. Typically, the symptoms of DTMUV infection include depression, delayed growth, and paralysis. Additionally, DTMUV can induce ovarian lesions in female ducks, resulting in a severe decrease of egg production ranging from 20% to 90%^5^. Consequently, DTMUV infection accounts for a great economic loss in the poultry industry in China. Therefore, effective strategies to prevent DTMUV infection are required.

Vaccination is one of the best methods to prevent DTMUV infection. In terms of genome and protein composition, DTMUV is a typical flavivirus. Its genome contains a unique open reading frame (ORF) that encodes three structural and seven non-structural proteins (NS). The structural proteins are core (C), pre-membrane/membrane (prM/M) and envelope (E) proteins^12^. Virions mature when the prM protein is cleaved to the M form by furin (a mammalian endopeptidase), which is located in the trans-Golgi network^13^. E protein is the major envelope protein of flaviviruses and contains three parts: an ectodomain, a stem region and a transmembrane domain^14,15^. These viruses contain abundant epitopes targeted by neutralizing antibodies. Thus, E protein is considered as the primary immunogen of flaviviruses^14,16^, and many flavivirus vaccines have been developed based on E protein^14,17^. To improve the immunity, efficient approaches are required for the proper expression, processing and secretion of E protein^18^. Truncated E protein lacking a membrane anchor region but maintaining an ecotodomain (the immunoepitope-enriched region) was utilized to increase the expression of secreted E protein^19^. Studies on tick-borne encephalitis have indicated that prM also plays an essential role in facilitating the secretion of E protein^20^. In addition, prM plays crucial roles in the folding, stability, and protective immunity development of E protein^20^. Partial protection is conferred when the vaccine only contains E protein without prM protein^21^, and complete protection against Zika virus is afforded when the vaccine contains prM with full-length, instead of truncated, E protein^22^. Additionally, the prM-E proteins of flaviviruses can self-assemble into a subviral particle that exhibits a similar structural feature and the same epitopes as the wild-type virion^23,24^. Therefore, several strategies have been employed to generate candidate DTMUV vaccines based on prM and E proteins. In these vaccination strategies, duck enteritis or Japanese encephalitis (JE) virus has been utilized as the vector for carrying *prM*-*E* genes^21,25^. Although these vaccines have yielded promising protection, they are still difficult to prepare for large-scale inoculation in clinical practice because of the high cost and complicated procedures. Additionally, the use of viruses as vectors might pose a risk of infectious virus contamination^25–27^. Thus, alternative vaccines that are easier to manipulate, safer to use and capable of eliciting protective immune responses against DTMUV are needed.

Since development, DNA vaccines with attractive features, including safe use and easy manipulation, have been of great interest for human as well as animal immunization. Indeed, DNA vaccines against flaviviruses have been developed in previous studies and verified as much safer than live attenuated and infectious clones-based vaccines^28^. However, a naked DNA vaccine may show low immunogenicity by traditional intramuscular or subcutaneous vaccination^29^. Many studies have demonstrated that the use of bacteria as functional carriers of DNA vaccines can compensate for this defect^30,31^. Live enteropathogenic bacteria, such as *Listeria monocytogenes*, *Salmonella* spp. and *Yersinia* spp., have previously been employed^32,33^. Among these bacteria, attenuated *S. typhimurium* has been demonstrated as an effective safe carrier and is consequently an extremely popular vector for delivering viral immunogenic genes^34,35^. Attenuated *S. typhimurium* as an intracellular bacterium can target M cells of mucosa-associated lymphoid tissue (MALT) via parenteral routes and subsequent transfer to the liver and spleen^30,36,37^. The internal DNA plasmids are released during host cell degradation, and the antigens are expressed under an eukaryotic promoter to stimulate specific immune responses against the pathogens^38^. Therefore, DNA vaccines carried by attenuated *S. typhimurium* have the potential to induce effective systemic immune responses with no additional equipment. Thus, this strategy is a promising choice for cost effective mass vaccination.

In the present study, we applied *S. typhimurium* aroA^−^ strain SL7207 as a vector to deliver recombinant plasmid DNA containing the coding sequences of *prM* and *E* genes, SME for short, of DTMUV. Neutralizing activities are associated with protection from flavivirus diseases^22,39^. Consistently, the present results showed that this oral vaccine induced high levels of neutralizing antibodies and conferred effective protection against DTMUV. In addition, attenuated *S. typhimurium* was verified to be an efficient DNA vaccine carrier for DTMUV in this study, providing a potential novel strategy for developing efficient vaccines against DTMUV.

## Results

### Expression of antigen proteins *in vitro*

**Figure 1:**
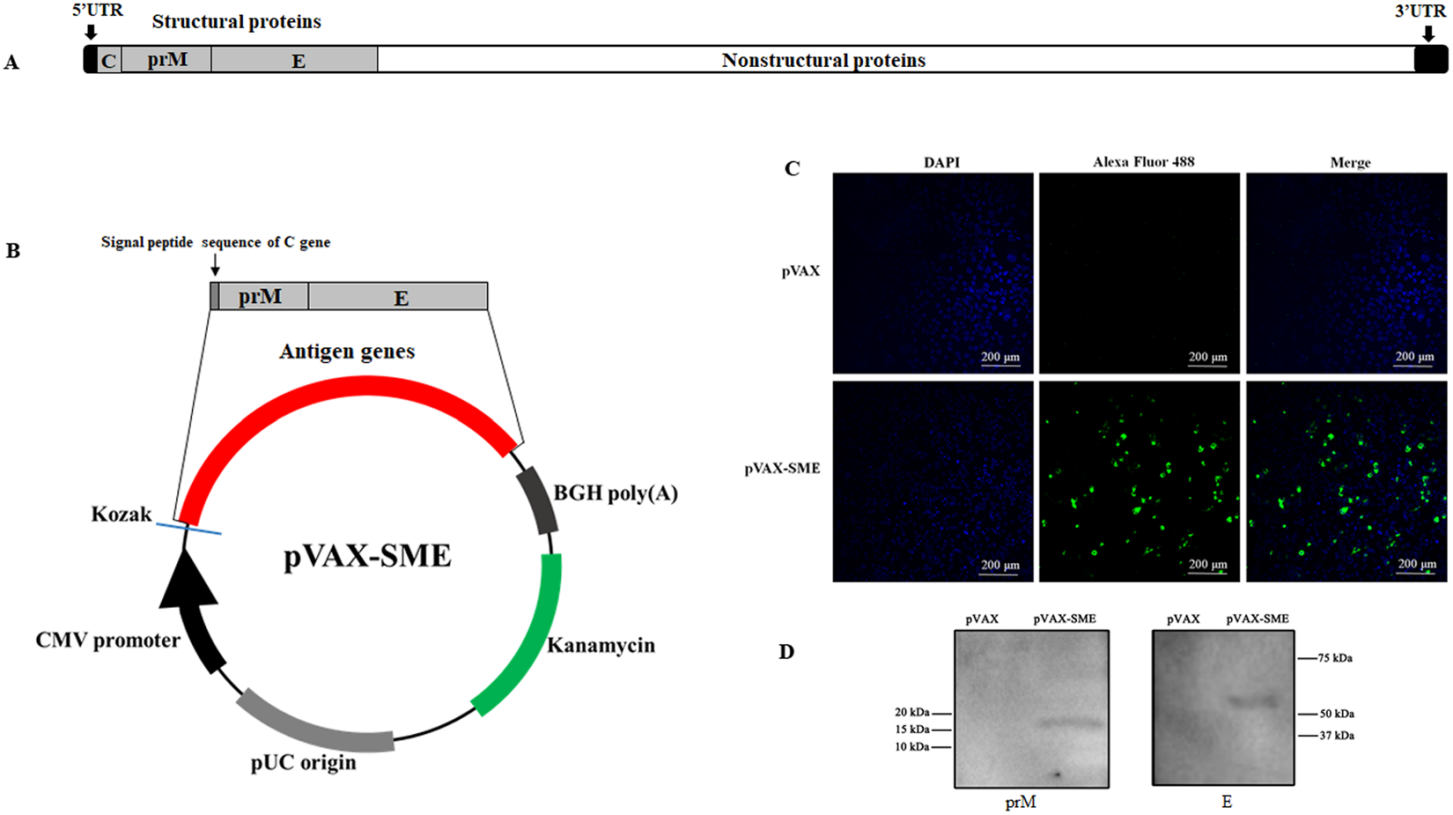
Expression of antigen genes *in vitro*. (**A**) Genomic structure of DTMUV, which includes genes encoding structural proteins (C, prM and E), nonstructural proteins, 5′-end untranslated region (5′UTR) and 3′UTR. (**B**) Genes encoding the structural proteins prM, E and the signal peptide of C proteins were cloned in to pVAX1 to generate the DNA vaccine plasmid pVAX-SME. **(C)** COS7 cells were transfected with plasmid pVAX or pVAX-SME. The expression of E protein was detected by indirect immunofluorescence assay using the rabbit anti-DTMUV-E protein polyclonal antibody and the Alexa Fluor 488-conjugated goat anti-rabbit IgG secondary antibody. Blue fluorescence indicates nuclei, and green fluorescence indicate DTMUV E proteins. **(D**) Duck embryo cells were transfected with plasmid pVAX-SME or pVAX. The expression of prM (left panel) and E (right panel) proteins was checked by Western blotting by using the mouse anti-DTMUV-prM polyclonal antibody combining horseradish peroxidase-conjugated goat anti-mouse antibody, or rabbit anti-DTMUV-E polyclonal antibody combining horseradish peroxidase-conjugated goat anti-rabbit antibody.

The DNA vaccine plasmid pVAX-SME was constructed by cloning the genes (2079 bp) encoding DTMUV envelope proteins (prM and E) and the signal peptide of DTMUV capsid protein (C) into the vector pVAX1 (Fig. 1A,B). A schematic map of the DNA vaccine plasmid is shown in Fig. 1B. To determine whether the antigen genes were successfully expressed from DNA vaccine plasmids, E proteins, which are encoded by the E gene located down-stream of the antigen gene cluster, were assessed by immunofluorescence assay in COS7 cells transfected with plasmid pVAX-SME or empty vector pVAX. As shown in Fig. 1C, specific green fluorescence, indicated that E proteins were observed in the cells transfected with pVAX-SME, whereas no fluorescence appeared in the cells transfected with empty vector pVAX (Fig. 1C). In addition, the expression of both prM and E antigen proteins *in vitro* was checked by Western blotting by using specific anti-prM or E protein antibody (Fig. S3). Both prM and E proteins are successfully expressed in the duck embryo cells transfected with the DNA vaccine plasmid pVAX-SME (Fig. 1D). These results suggested that the DNA vaccine plasmid was successfully constructed and both prM and E antigen genes were efficiently expressed from the DNA vaccine plasmid *in vitro*.

### Expression of antigen *in vivo*

**Figure 2:**
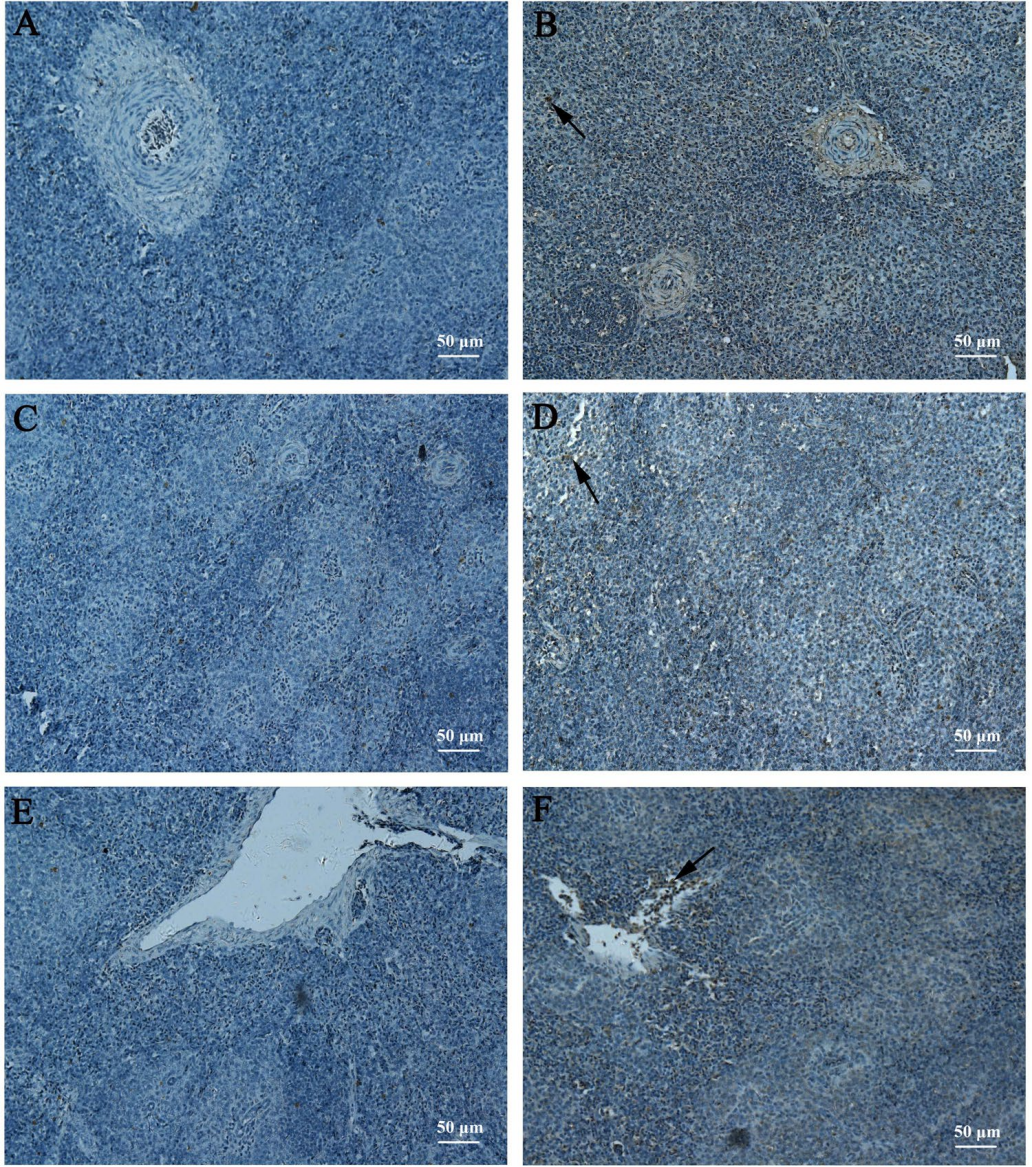
Expression of E protein *in vivo*. Spleens from each group were collected at 3 days post-initial immunization (n = 3). E protein in spleen was detected by immunochemistry. Spleen slides from groups that vaccinated with PBS **(A)**, inactivated DTMUV vaccine **(B)**, empty vector pVAX **(C)**, pVAX-SME vaccine **(D)**, SL7207 (pVAX) **(E)** and SL7207 (pVAX-SME) vaccine **(F)** were stained with the rabbit polyclonal antibody against DTMUV E protein primary antibody and the horseradish peroxidase-conjugated goat anti-rabbit secondary antibody. Arrows indicate the E proteins.

To verify the expression of DTMUV-E protein *in vivo*, immunohistochemistry assay of spleens collected from different vaccinated groups at 3 days post-immunization was performed. As shown in Fig. 2, specific brown spots which represented the DTMUV-E protein antigen was observed on the slides from ducks in groups intramuscularly vaccinated with the inactivated DTMUV (Fig. 2B), pVAX-SME (Fig. 2D) as well as the group orally inoculated with the DNA vaccine SL7207 (pVAX-SME) (Fig. 2F), whereas an absence of brown spots on the slides of negative control groups PBS (Fig. 2A), pVAX (Fig. 2C) and SL7207 pVAX (Fig. 2E) was observed. These observations indicate that the antigen E protein gene in the constructed DNA vaccine plasmid pVAX-SME was successfully expressed *in vivo*. Moreover, the DNA vaccine orally delivered by using attenuated *S. typhimurium* SL7207 was an efficient and successful inoculation method.

### Immunogenicity of the vaccines

**Figure 3:**
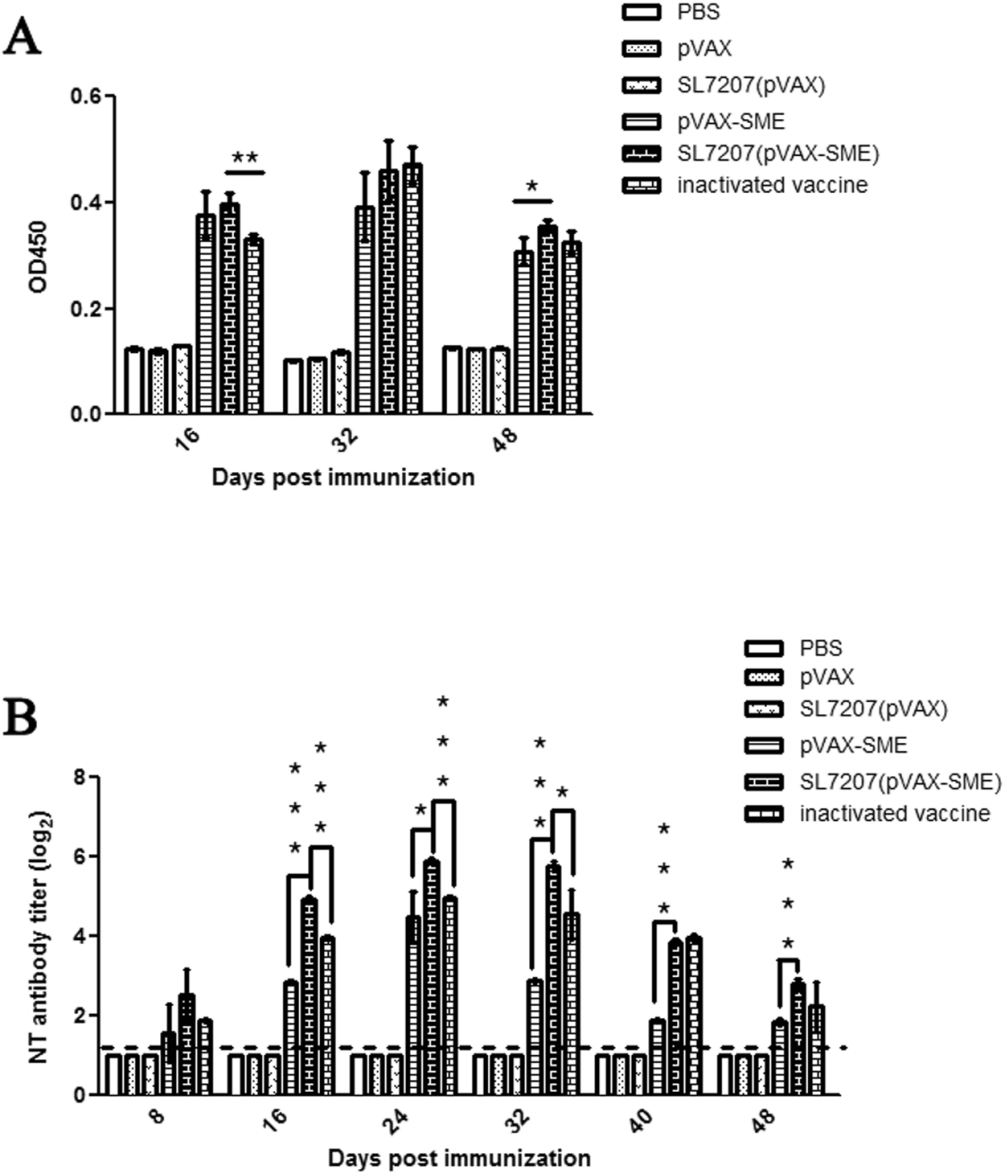
Production of specific antibody *in vivo* induced by the oral DNA vaccine. (**A)** The sera IgY antibodies specific to DTMUV E protein were checked by ELISA. The collected serum samples were diluted and incubated with the E protein coated plate. The specific anti-DTMUV-E protein antibodies were measured by horseradish peroxidase-conjugated goat anti-bird IgY. The levels of the specific antibodies are shown as the means ± standard deviations (n = 3 of each time point). **(B)** Neutralizing (NT) antibodies against DTMUV in the serum were detected by neutralizing assay. The titers of the neutralizing antibodies against DTMUV were detected and presented as the log2 changed folds (Y-axis) reference to the negative control PBS, pVAX and SL7207 (pVAX) groups. Data are shown as the means ± standard deviations (n = 3 of each time point). The dash line indicates the lowest threshold value for positive reaction in the neutralizing assay. In addition, significant higher titers of the neutralizing antibodies was observed in SL7207 (pVAX-SME) vaccinated group compared to that in pVAX-SME or inactivated vaccine groups (^*^p < 0.05, ^**^p < 0.01, ^***^p < 0.001). All data were graphed by GraphPad Prism v.5.0 (La Jolla, CA, USA).

To evaluate the immunogenicity of the developed oral DNA vaccine, a specific antibody IgY in serum against DTMUV E protein, which is a major humoral antibody in birds^40^, was analyzed in different vaccinated groups by using indirect ELISA. As shown in Fig. 3A, similar to groups that intramuscularly vaccinated with naked pVAX-SME and inactivated DTMUV, a high level of specific antibodies against E protein in the oral vaccine SL7207 (pVAX-SME) group was detected, which showed approximately a 4-fold higher level compared to the negative control PBS, pVAX and SL7207 (pVAX) groups (Fig. 3A). The titers of the antibodies in the SL7207 (pVAX-SME) group remained at a high and stable level from 16 to 48 dpi and was even slightly higher compared to that in inactivated vaccine group at 16 dpi and the pVAX-SME group at 48 dpi (Fig. 3A). These results suggested that prM and E antigen-carrying DNA vaccines efficiently induced specific immune responses *in vivo* similar to the inactivated DTMUV vaccine. In addition, the oral SL7207 (pVAX-SME) DNA vaccine, delivered by bacteria, was an efficient strategy to vaccinate ducks to fight against DTMUV.

### Neutralizing antibodies responses

To verify the protective immune response induced by the developed vaccine, neutralizing activity against the DTMUV virus from the serum was detected in different vaccinated groups by using a neutralizing assay. As shown in Fig. 3B, the neutralizing antibodies against DTMUV were significantly detected in the group orally inoculated with SL7207 (pVAX-SME) as well as the groups that intramuscularly vaccinated with naked pVAX-SME and inactivated DTMUV vaccines. The antibodies were detected at 8 days after the first immunization, and the titers of the antibodies gradually increased, peaking at 24 days post-immunization with approximately 6 log2-folds, 4.5 log2-folds and 5 log2-folds higher in the SL7207 (pVAX-SME), pVAX-SME and inactivated DTMUV groups respectively compared to the threshold value of positive reaction. Among these three groups, the highest titers of antibodies were observed from 8 to 48 days post-vaccination in the oral SL7207 (pVAX-SME) vaccine group, followed by the inactivated DTMUV and pVAX-SME groups. There were no neutralizing antibody responses in ducks from the negative control vaccine groups during entire experimental period (Fig. 3B). Altogether, the results herein indicated that the developed oral SL7207 (pVAX-SME) DNA vaccine efficiently induced the production of a high titer of neutralizing antibodies against DTMUV *in vivo*.

### Protection of ducks against DTMUV challenge

**Figure 4:**
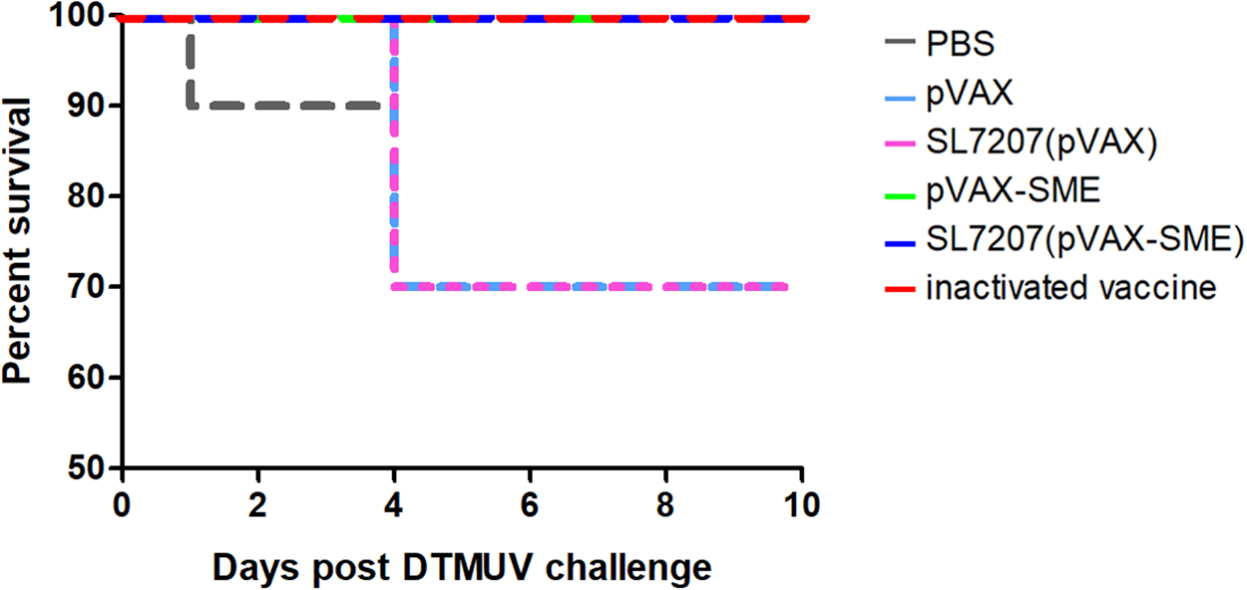
Survival curves post challenge with lethal dose of virulent DTMUV. The immunized ducks (n = 10/group) were challenged with 10^4.5^ ELD_50_ (6 × 10^6^ PFU) DTMUV at 16 days after the second immunization. The death number of ducks was recorded for consecutive 10 days after virus challenge and graphed by GraphPad Prism v5.0.

To verify the protective ability of the developed oral DNA vaccine against DTMUV infection, vaccinated ducks were challenged with 10^4.5^ 50% ELD_50_ (6 × 10^6^ PFU) DTMUV at 16 days after the second vaccination. As shown in Fig. 4, 30% of the ducks in the PBS, pVAX and SL7207 (pVAX) groups died at the end of the experiment; however, all of the ducks in the SL7207 (pVAX-SME)-immunized group survived, similar to the animals in the pVAX-SME and inactivated vaccine groups. Thus, the survival ratios in all PBS, pVAX and SL7207 (pVAX) negative control groups were 70%, while the survival ratio in SL7207 (pVAX-SME), pVAX-SME and inactivated vaccine groups was 100%. In addition to comparing the mortality between the vaccine and control groups, the clinical signs of the ducks were recorded to evaluate the efficacy of the developed DNA vaccine. The typical clinical signs of DTMUV infection, such as depression, inappetence and reluctance to move, were observed in the surviving ducks in the PBS, pVAX and SL7207 (pVAX) groups in the next two weeks after challenge. However, these clinical signs were only slightly observed in a few of the ducks in the SL7207 (pVAX-SME), inactivated DTMUV vaccine and pVAX-SME groups during the early stage (1 to 3 days after challenge) of the challenge experiment, and these ducks recovered soon. These results suggested that the oral SL7207 (pVAX-SME) DNA vaccine efficiently protected the ducks against the DTMUV infection.

## Discussion

DTMUV, a newly emerged avian flavivirus, is important for the poultry industry and may have an impact on public health^2,41^. Thus, vaccine development is urgently needed. Although protection against DTMUV has been achieved in previous studies, there is still much room for further improvement. Conventional attenuated DTMUV vaccines may pose the risk of reversible virulence and creation of new virus strains. Chimeric DTMUV vaccines constructed by using reverse genetics may fail to attenuate virulence of DTMUV in mice tests^25^. Purified envelope proteins combined with adjuvant is a safer option, but these vaccines are impossible for large-scale inoculations in clinical practice because of the high cost and intricate delivery methods^17^. DNA vaccines have several advantages, such as safety, simple manufacture, low cost and biological stability^42,43^. In addition, the immune responses of DNA vaccines can be enhanced by the improvement of DNA delivery^32,34^. Considering these findings, a DTMUV DNA vaccine expressing prM and E proteins delivered by attenuated *S. typhimurium* SL7207 for oral inoculation was designed in the present study. Here, the SL7207 (pVAX-SME) vaccine induced high levels of humoral immunity and completely protected ducks from lethal DTMUV challenge. These results indicated that the prM and E proteins were efficiently expressed in vaccinated ducks through oral inoculation, and the envelope proteins, prM and E were sufficient to elicit substantial antibodies to protect ducks against DTMUV. These conclusions are consistent with those of previous works on other flaviviruses^22,44^. Moreover, compared with the intramuscular injection with DNA vaccine pVAX-SME, we demonstrated that the oral DNA vaccine can elicit more robust neutralizing antibodies, and the ducks vaccinated with SL7207 (pVAX-SME) had no clinical observations post-DTMUV challenge. Hence, the oral DNA vaccine SL7207 (pVAX-SME) is more promising than the i.m. DNA vaccine pVAX-SME.

It has been reported that the vaccine based on nucleocapsid protein of Dengue virus is able to provide immune-protection, but without inducing the production of antibody^45^. This observation makes the capsid protein an attractive vaccine candidate against flavivirus since it eliminates the potential risk of the induction of antibody dependent enhancement (ADE) of infection, which is a natural phenomenon observed on Dengue virus infection: a specific range of antibody titers enhance viral replication *in vitro* and severe disease in animal models^46^. Thus, in addition to the structural proteins of prM and E, the capsid (C) protein of DTMUV has also been applied for developing a similar oral DNA vaccine in our previous study^47^. However, different from the capsid protein of Dengue virus, the DTMUV-C protein based vaccine not only induced immune-protection but also induced the production of neutralizing antibody^47^. The reasons for the different behaviors caused by DTMUV-C protein and Dengue virus capsid protein are still unclear, but DTMUV-C protein has been shown to lose its advantage in the development of a DTMUV vaccine. Besides, the level of neutralizing antibody induced by DTMUV-C protein^47^ is slightly lower than that by prM and E proteins in this study (Fig. 4B). Thus, we assume utilization of prM and E protein as an antigen is the most promising strategy for developing a vaccine against DTMUV infection so far.

Neutralizing antibodies elicited by vaccines are associated with protection from flavivirus infection^39,44^. A proper signal sequence is needed for *prM*-*E* genetic vaccines to achieve high levels of neutralizing antibodies^48^. There is barely neutralizing activity against DTMUV using duck enteritis virus as a vector to deliver *prM*-*E* genes without any signal sequence^21^. In flaviviruses, the C-terminus of the C protein is a signal sequence to mediate the translocation of prM into the lumen of the endoplasmic reticulum (ER)^49^. Previous studies have reported that flavivirus DNA vaccines expressing prM-E with the C-terminus of C protein could enhance the production and secretion of envelope subvirion particles^50,51^. Thus, the C-terminus of the DTMUV *C* gene was employed as a signal sequence fused with *prM*-*E* in the present DNA vaccine, and the results demonstrated that this DNA vaccine could induce high levels of anti-DTMUV neutralizing antibodies during the entire experiment. These results suggested that the C-terminus of C protein may be a functional signal sequence that enhances the expression and secretion of DTMUV envelope proteins.

To make the DTMUV vaccine easier to use in clinical practice, attenuated *S. typhimurium* SL7207 was used as a carrier to deliver SME genes by oral administration in the present study. Attenuated *S. typhimurium*-based DNA vaccine is considered an ideal vaccine possessing several advantages and has been extensively studied in anti-viral research in the poultry industry^32,52^. First, *Salmonella*, as a Gram-negative bacterium, contains lipopolysaccharide (LPS), which can be recognized by Toll-like receptor 4 and thereby induce immune activation, has been demonstrated as a potent adjuvant^53,54^. Thus, using attenuated *S. typhimurium* as a vector can enhance antigen-specific immune responses^53,55^. Second, *S. typhimurium* is an intracellular pathogen that can deliver antigens through natural infective routes, such as spray, oral and intranasal inoculation. Therefore, DNA vaccines delivered by the *S. typhimurium* vector are convenient to prepare and thereby can be conducted as a massive vaccination in the poultry industry. DNA plasmids are released when attenuated *S. typhimurium* are degraded and migrate to the nucleus of host cells where the exogenous antigen can be transcribed, translated and posttranslationally modified^56^. The present study provides evidence supporting these hypotheses through the development of a *Salmonella*-based DNA vaccine delivery system. Although considerable amounts of antibodies were generated by the intramuscular injection of pVAX-SME vaccine, higher levels of antibodies were presented in ducks by oral immunization with the SL7207 (pVAX-SME) vaccine. Additionally, and surprisingly, the neutralizing activities in ducks from the inactivated vaccine group were even weaker than those from the SL7207 (pVAX-SME) vaccine group. These results indicated that SL7207 could enhance the immune responses against DTMUV. Potential mechanisms are associated with the adjuvant activity of *S. typhimurium* and/or SME genes were more efficiently expressed in professional APCs through *S. typhimurium* delivery. Ducks, intramuscularly injected with pVAX-SME vaccine, showed certain clinical pathological features, such as apathetic symptoms and appetite loss at early time periods (1 to 4 days after lethal DTMUV challenge). Conversely, these pathological features were not observed in ducks immunized with SL7207 (pVAX-SME) vaccine, and all of these animals survived the lethal DTMUV infection. Collectively, these results showed that the DTMUV DNA vaccine delivered by attenuated *S. typhimurium* was an efficient strategy to achieve high titers of neutralizing antibodies and confer efficient protection of ducks from lethal DTMUV infection.

Taken together, in the first study of DTMUV DNA vaccines, we have demonstrated that SME cassette possesses good immunogenicity, as the results indicated that SME genes inserted into the pVAX1 plasmid could confer effective protection to ducks against lethal DTMUV challenge either through intramuscular or oral immunization. Moreover, the SL7207 (pVAX-SME) vaccine induced higher levels of neutralizing antibodies than pVAX-SME and inactivated DTMUV vaccines did. Therefore, it is obvious that attenuated *S. typhimurium* could be an efficient delivery vector, and the SL7207 (pVAX-SME) vaccine could be a candidate vaccine against DTMUV. The present study provides a new insight into the development of a commercial DTMUV vaccine.

## Materials and Methods

### Ethics and Bio-containment statement

The animal studies were approved by the Institutional Animal Care and Use Committee of Sichuan Agricultural University (29^th^ 2014, Permit Number: SYXK 2014-187), China. All methods used in animal experiments were carried out in accordance with the relevant guidelines and regulations of National Institutes of Health. The DTMUV virus-related experimental manipulation was performed inside a bio-containment device in the BSL-2 facility.

### Strains, virus, cell line and animals

The attenuated *Salmonella typhimurium* aroA^-^ strain SL7207 (*S. typhimurium* 2337-65 derivative hisG46, DEL407 [*aroA*::Tn10 (Tc^s^)]) was kindly provided by Professor Kai Schulze of Helmholtz Center for Infection Research (Germany). DTMUV (GenBank: JX196334.1) was generously provided by Professor Yu Huang, Fujian Academy of Agricultural Sciences (China). The virus was propagated in the allantoic cavities of 9-day-old specific pathogen-free (SPF) embryonated duck eggs, and the virus suspension was stored at −80 °C until further use. DTMUV-free shelducks (one-day-old) were purchased and fed under standard conditions.

### Preparation of inactivated DTMUV vaccine

The DTMUV inactivated vaccine was prepared as previously described^57^. Briefly, the DTMUV (GenBank: JX196334.1) was propagated in the allantoic cavities of 9-day-old specific pathogen-free (SPF) embryonated duck eggs. The allantoic fluid containing DTMUV was collected at 4 days post-inoculation. The supernatant of virus suspension was obtained by centrifugation at 4 °C, 6000 r/min for 15 min and stored at −80 °C until further use. The virus titer was assessed in duck embryo fibroblasts (DEF) cells by using a plaque assay. The virus suspension (6 × 10^6^ PFU per ml) was inactivated by incubation with 0.2% formaldehyde (v/v) at 37 °C for 24 h. The inactivated viruses were mixed with an equal volume of aluminum adjuvant and emulsified by using emulsification mixer (Fluko, China) to generate the inactivated DTMUV vaccine.

### Construction of recombinant DTMUV DNA vaccines

Total RNA was extracted from allantoic fluid containing DTMUV by Trizol (Invitrogen, USA) and reverse transcribed into cDNA by random primers (Takara, Japan) according to the manufacturer’s instructions. The SME genes including C-terminus of *C* gene (GenBank: JX196334.1) was amplified with primers (Fw-TACAGAATTCACTATGGCACGGAAAGCGAAACGTCGGGGGGG, Rev TACACTCGAGCTAGGCATTGACATTTACTGCCAGGAAGACTAAAATTCC) from cDNA template and cloned into the multiple cloning site of pVAX1 (Invitrogen, USA) vector by using *Eco*RI and *Xho*I enzymes to generate the DNA vaccine plasmid pVAX-SME. The plasmid was confirmed by digestion test (Supplementary Information Fig. S1) and DNA sequencing. The pVAX-SME or pVAX was transformed into attenuated *S. typhimurium* SL7207 by electroporation^35^ to generate SL7207 (pVAX-SME) and SL7207 (pVAX), respectively.

### Indirect immunofluorescence

COS7 cells were transiently transfected with DNA vaccine plasmid pVAX-SME or vector pVAX by using Lipofectamine 2000 (Invitrogen, USA) when the monolayer cells grown in 6-well plates reached to approximately 80% confluent. After incubation for 48 h, the cells were fixed with 4% paraformaldehyde for 15 min at room temperature and then permeabilized with 0.2% Triton X-100 in PBS for 10 min at room temperature. Then, the expression of *E* gene located down-stream of the antigen gene cluster of the DNA vaccine was assessed by an indirect immunofluorescence assay as previously described^35^. Briefly, the cells were blocked in 5% BSA buffer for 1 h at room temperature and then incubated with 1:100 diluted rabbit anti-E polyclonal antibody (prepared in our lab) at 4 °C overnight. After washing three times with PBS, the cells were incubated with 1:2000 diluted Alexa Fluor 488-conjugated goat anti-rabbit IgG antibody (Thermo Fisher, USA) in a dark room at room temperature for 2 h. The cells were then washed four times with PBS and counterstained with 4′,6-Diamidine-2′-phenylindole dihydrochloride (DAPI) for 10 min at room temperature. Then, the cell fluorescence was examined and imaged by fluorescence microscopy (Nikon, Japan).

### Western blot analysis

For Western blotting, duck embryo cells were transiently transfected with DNA vaccine pVAX-SME or vector pVAX as described above. At 48 h after transfection, the cells were collected and resuspended in cell lysate buffer (20 mM Tris, pH 7.4, 150 mM NaCl, 1% Triton X-100, 0.1% SDS, 1 μg aprotinin, 1 mM phenylmethanesulfonyl fluoride). A total of 20 μg of the lysate was separated by SDS-PAGE and transferred to polyvinylidene difluoride (PVDF) membranes by using a Trans-Blot Semi-Dry Transfer Cell (Bio-Rad, USA) according to the manufacturer’s instructions. The membranes were then incubated with 5% skim milk in phosphate buffer (pH 7.2) containing 0.05% Tween-20 (5% milk-PBST) at 4 °C overnight. Then, the membrane was incubated with the primary antibody (mouse anti-DTMUV-prM polyclonal antibody or rabbit anti-DTMUV-E polyclonal antibody (prepared by our lab) diluted 1:20 in 5% milk-PBST) at room temperature for 1 h, followed by three 10-min washing steps with PBST. Then, the membrane was incubated for 1 h with the secondary antibody (horseradish peroxidase-conjugated goat anti-mouse or goat anti-rabbit IgG (Transgen Biotech, China) diluted 1:2000 in 5% milk-PBST). After three washing steps with PBST, the membrane was detected with ChemiDoc^TM^ (Bio-Rad, USA) by using Immun-Star^TM^ HRP (Bio-Rad, USA) as chemiluminescence substrate.

### Immunohistochemical analysis

Ducks in each vaccinated group were euthanized at 3 days post-first immunization, and the spleens were collected (n = 3). The expression of the E protein was confirmed by immunohistochemistry as previously described^58^. Briefly, the tissues were cut into section at a 4 μm thickness (Leica RM2128, Germany) after fixation with 4% paraformaldehyde and embedding in paraffin. The sections were dewaxed with xylene, re-hydrated with gradient ethanol and blocked with 0.3% hydrogen peroxide (H_2_O_2_), and the antigen retrieval was performed with citrate buffer solution (CBS, 0.01 M, pH 6.0). Then, the sections were incubated with 10% normal goat serum to block the unspecific antigens for 1 h at 37 °C. Subsequently, the sections were stained with a rabbit polyclonal antibody against DTMUV E protein (prepared in our lab) in 1:200 dilution overnight at 4 °C and a horseradish peroxidase-conjugated goat anti-rabbit antibody (Transgen Biotech, China) at a 1:1000 dilution for 2 h at room temperature. The expression of the E protein was visualized with DAB (Solarbio, China) and graphed by microscopy (Olympus BX43, Japan).

### Enzyme-linked immunosorbent assay (ELISA)

Sera samples were collected at different time points as described in Supplementary Information (Fig. S2). The presence of the specific anti-DTMUV E protein IgY antibody from the serum in vaccinated ducks was examined by using indirect ELISA. To this end, 100 µl of purified E proteins (1 µg/ml) as a capture molecular was incubated in 96-well ELISA plates at 4 °C overnight. The plates were washed three times with PBST and blocked by 1% BSA for 1 h at room temperature. Sera samples were added 100 µl per well at a dilution of 1:400 (n = 3 of each time point) and incubated for 1 h at 37 °C. Then, the plates were washed three times with PBST. Horseradish peroxidase conjugated goat anti-bird IgY (Abcam, UK) was used to detect bound antibodies with 1:2000 dilution for 1 h at 37 °C. The plates were visualized with 3,3′,5,5′-tetramethy1 benzidine (TMB) in the dark for 10 min at room temperature, and the reaction was stopped with 2 M H_2_SO_4_. Finally, the reaction was measured under the OD at 450 nm. Each sample was independently measured two times.

### Neutralization assay

Sera were collected and submitted for neutralizing antibodies assay as described previously^21^. Sera samples were inactivated in 56 °C water bath for 30 min (n = 3 of each time point) and serial 2-fold diluted in Minimum Essential Media (Thermo Fisher). Each sample was mixed with an equal volume of 100-fold 50% tissue culture infective dose (TCID_50_) of DTMUV and incubated in 96-well plates at 37 °C for 1 h. BHK-21 cells then were seeded onto each well and co-incubated with the mixture for 5 days. Titers of neutralizing antibodies were determined by monitoring the cytopathic effect (CPE). Neutralizing activity was recorded until two out of three wells of infected cells showed no CPE.

### Vaccination and challenge experiments

The 168 ducks at 7 days old were randomly divided into 6 groups with 28 ducks each. The vaccination was performed in ducks at 8 days old. One group of ducks was intramuscularly injected in the lateral thigh with 0.5 ml of PBS. Two groups of ducks were intramuscularly injected in the lateral thigh with vector pVAX or vaccine pVAX-SME at a dose of 200 μg in 0.5 ml of PBS. Two groups of ducks were orally inoculated with vector SL7207 (pVAX) or vaccine SL7207 (pVAX-SME) at a dose of 10^10^ CFU in 0.5 ml of PBS. The last group of ducks was intramuscularly injected with 0.5 ml of inactivated DTMUV vaccine at lateral thigh. Ducks from each group were immunized the second time at an interval of 16 days with the same doses of vaccine as used in the primary injection. The details of the immunization schedule are provided in Supplementary Information (Fig. S2). Ten ducks from each group were randomly selected and challenged with lethal DTMUV at a dose of 10^4.5^ ELD_50_ (6 × 10^6^ PFU) by intravenous injection at 16 days post-second immunization. The clinical symptoms and death of those challenged ducks was checked and recorded for continuous 10 days afterwards. The details of the challenge are described in Supplementary Information (Fig. S2).

### Statistical analysis

The titers of antibody in ELISA assay and neutralizing assay were compared between different vaccinated groups. Statistical differences were assessed by using unpaired two-tailed Student’s t-test. Survival statistics utilized the log-rank test. Data were expressed as the means ± standard deviation. A significant difference was considered when the p value was less than 0.05. Statistical significance was set at ^*^p < 0.05, ^**^p < 0.01 and ^***^p < 0.001. All analyses were performed by using GraphPad Prism v.5.0 (La Jolla, CA, USA).

### Data availability

The data generated during the present study are available from the corresponding author upon reasonable request.

## Electronic supplementary material

Supplementary Information
