# Supplementary material for: Oral Delivery of a DNA Vaccine Expressing the PrM and E Genes: A Promising Vaccine Strategy against Flavivirus in Ducks
Source: Sci Rep. 2018 Aug 17;8:12360. doi: 10.1038/s41598-018-30258-3 (PMC6098003; doi:10.1038/s41598-018-30258-3)
Supplement: Supplementary file 1 — Supplementary Information [file 41598_2018_30258_MOESM1_ESM.pdf]

Supplementary Information

Oral Delivery of a DNA Vaccine Expressing the PrM and E Genes: A Promising Vaccine Strategy against Flavivirus in Ducks

Juan Huang[1,2,3,#], Renyong Jia[1,2,3,#,*], Haoyue Shen[1,2,3,#], Mingshu Wang[1,2,3], Dekang Zhu[1,2,3], Shun Chen[1,2,3], Mafeng Liu[1,2,3], Xinxin Zhao[1,2,3], Ying Wu[1,2,3], Qiao Yang[1,2,3], Zhongqiong Yin[3], Anchun Cheng[1,2,3,*]

1 Avian Disease Research Centre, Sichuan Agricultural University, Chengdu 611130, China
2 Institute of Preventive Veterinary Medicine, Sichuan Agricultural University, Chengdu 611130, China
3 Key Laboratory of Animal Disease and Human Health of Sichuan Province, Chengdu, Sichuan 611130, China
# These authors contributed equally to this work
* Corresponding authors: jiary@sicau.edu.cn, chenganchun@vip.163.com

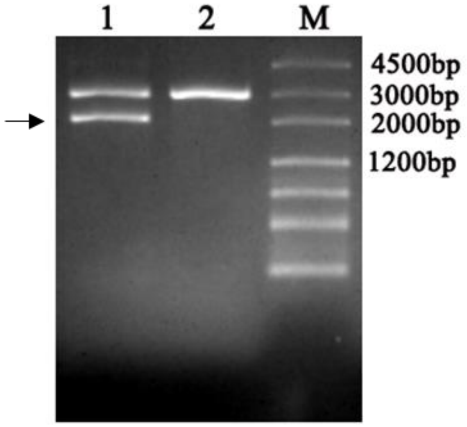

Figure S1. Digestion test of the constructed DNA vaccine plasmid pVAX-SME. The constructed DNA vaccine plasmid pVAX-SME (lane 1) and pVAX (lane 2) was digested by restriction enzymes *Eco*RI and *Xho*I. M represents DNA marker. The DNA fragment (2079 bp) of tandem prM-E antigen gene from pVAX-SME was indicated by the black arrow.

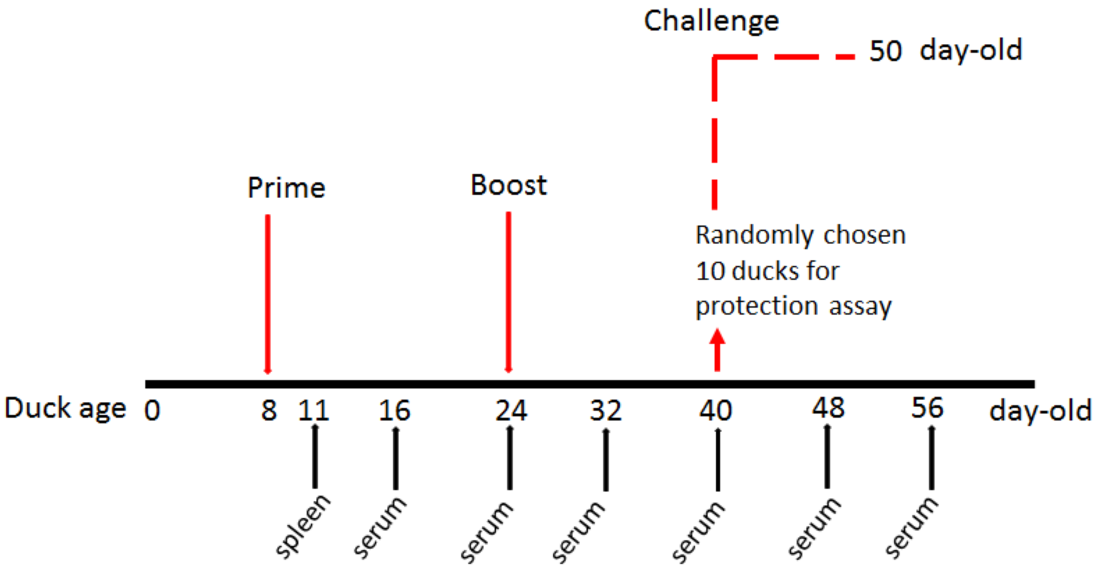

Figure S2. Schedules of vaccination and sample collection. Red arrows indicate the time points of primary and boost immunization. Red dash line represent 10 ducks from each vaccinated group are randomly chosen and challenged with $10^{4.5}$ ELD50 lethal DTMUV. The clinical symptoms and death of those challenged ducks was checked and recorded for continuous 10 days afterwards. Black arrows indicate the time points of specimen collection.

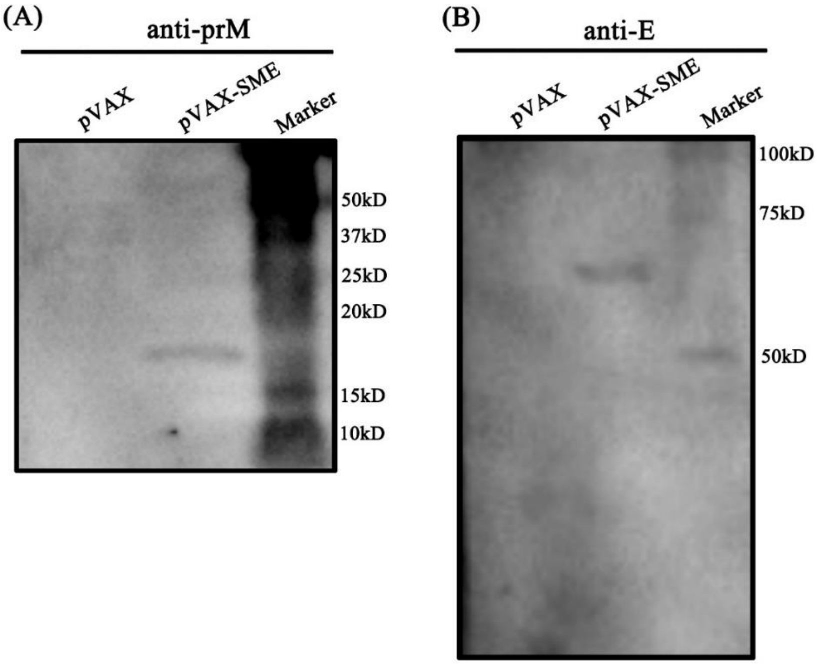

Figure S3. Western blot analysis of antigen expression. Duck embryo cells were transfected with plasmid pVAX-SME or pVAX. The expression of prM **(A)** and E **(B)** proteins was checked by Western blotting by using the mouse anti-DTMUV-prM polyclonal antibody combining horseradish peroxidase-conjugated goat anti-mouse antibody, or rabbit anti-DTMUV-E polyclonal antibody combining horseradish peroxidase-conjugated goat anti-rabbit antibody.
